# When the Body Speaks and the Tone Echoes: Exploring GAI Multimodal Emotion Recognition

**DOI:** 10.1192/j.eurpsy.2026.11357

**Published:** 2026-07-31

**Authors:** H. Dery, D. Piterman, Z. Elyoseph, E. Refoua, G. Meinlschmidt, K. Bar, D. Hadar Shoval, A. Geller

**Affiliations:** 1 School of Counseling and Human Development, University of Haifa, Haifa, Israel; 2 Department of Brain Science, Imperial College, London, United Kingdom; 3 Department of Psychology, Bar-Ilan University, Ramat-Gan, Israel; 4 Department of Psychology, Trier University, Trier, Germany; 5 Department of Digital and Blended Psychosomatics and Psychotherapy, University of Basel and University Hospital Basel, Basel, Switzerland; 6 School of Computer Science, Reichman University, Herzliya; 7 Department of Psychology and Educational Counseling, Max Stern Yezreel Valley College, Emek Yezreel; 8 Ruppin Academic Center, Emek Hefer, Israel

## Abstract

**Introduction:**

This study examined the social-cognitive capabilities of three Generative Artificial Intelligence (GAI) models from the Gemini family—Gemini Pro 1.5, Gemini Pro 2, and Gemini Flash 2—focusing on their ability to recognize human emotions from nonverbal cues, specifically vocal tone and bodily gestures. Grounded in mentalization theory—the capacity to attribute mental states to oneself and others, which underpins adaptive social functioning and therapeutic change—the research investigated the extent to which GAI models can approximate human-like emotion recognition.

**Objectives:**

The research investigated the extent to which GAI models can approximate human-like emotion recognition from nonverbal channels and whether the accuracy of this recognition is influenced by emotional valence (positive vs. negative).

**Methods:**

The study employed a comparative experimental design. Stimuli were drawn from the validated EU-Emotion Stimulus Set (O’Reilly et al., 2016) and EU-Emotion Voice Database (Lassalle et al., 2019). The dataset included 36 dynamic video clips representing bodily gestures and 42 audio recordings conveying vocal expressions. The GAI models were evaluated using zero-shot prompting under standardized conditions. Altogether, the study comprised 9,360 independent trials across both modalities. Human accuracy benchmarks were derived from the normative validation data of the EU-Emotion datasets, allowing for direct and controlled comparisons between model and human performance.

**Results:**

The findings revealed both modality- and valence-dependent patterns. GAI models demonstrated performance comparable to human participants in vocal emotion recognition; Gemini Pro 2 achieved an accuracy rate of 47.14%, closely matching the human benchmark of 45.19%. In contrast, the models underperformed in the bodily gesture modality, where human participants achieved 77.08% accuracy, whereas Gemini Pro 2 reached 71.39%. A consistent valence-based performance bias was observed: all GAI models demonstrated significantly higher accuracy for positive emotions than for negative ones, particularly in the bodily modality. In contrast, human participants showed no significant difference in recognition accuracy for positive and negative emotions. All models consistently performed better with bodily gestures than with vocal expressions.

**Image 1:**
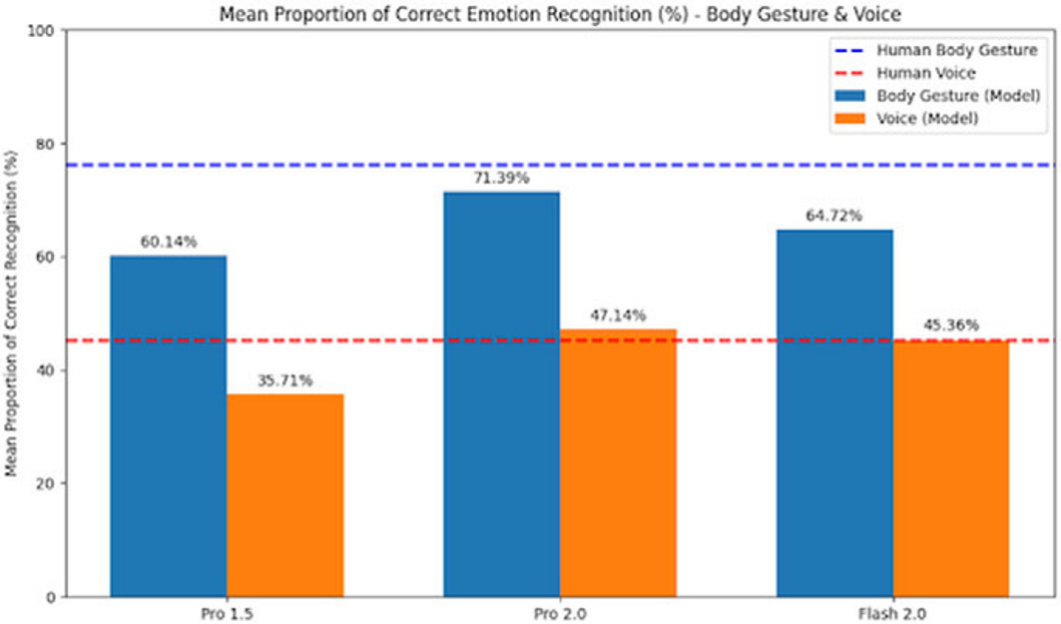
Image 1: Long description.

**Image 2:**
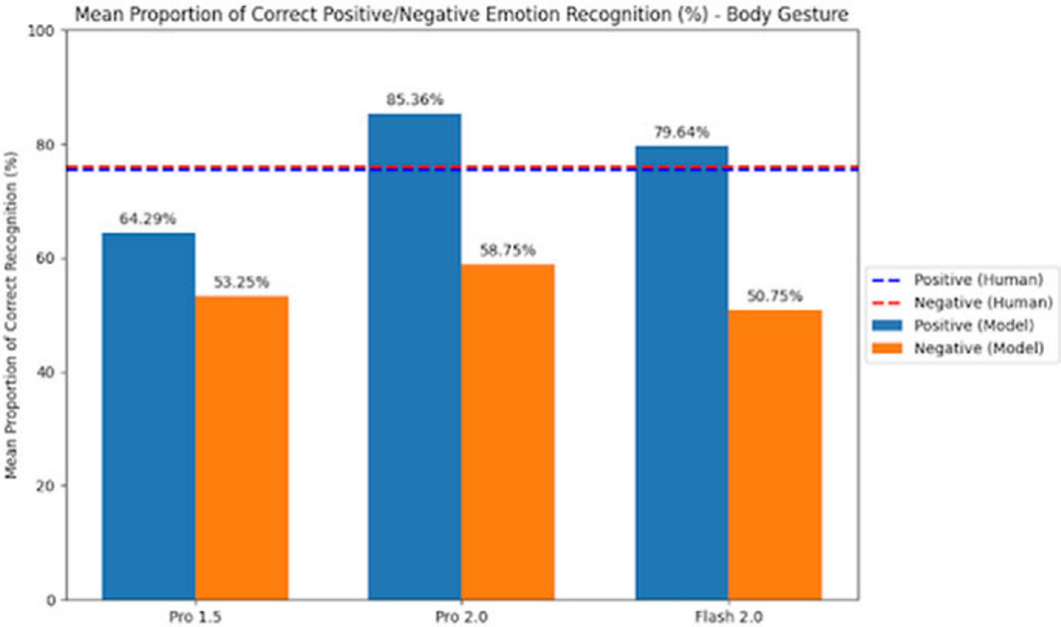
Image 2: Long description.

**Image 3:**
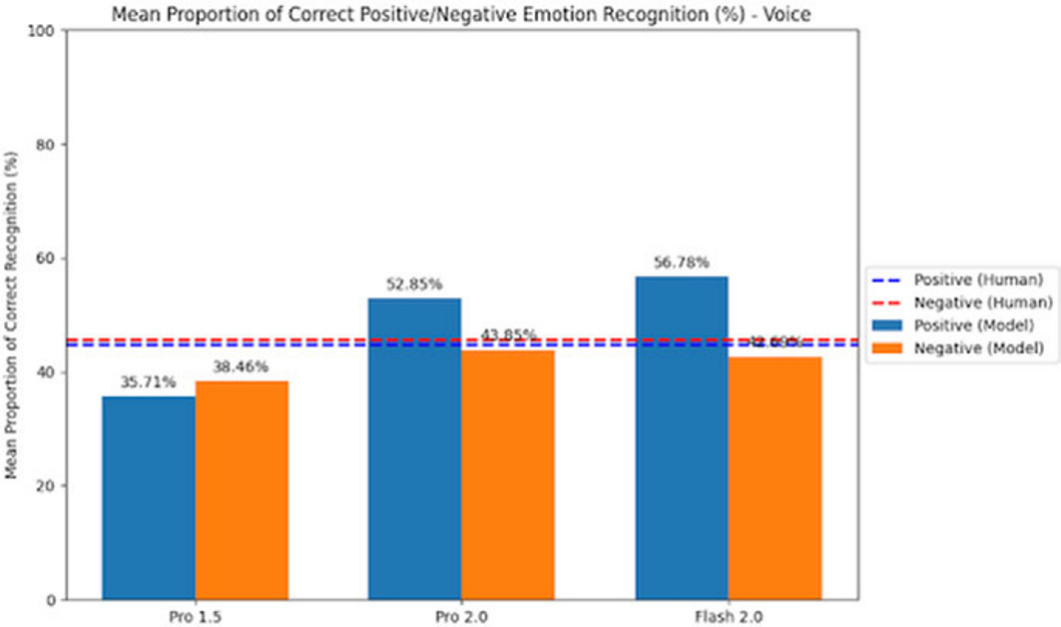
Image 3: Long description.

**Conclusions:**

This study provides detailed empirical evidence of GAI’s present capacity for nonverbal emotion recognition. While some models—particularly Gemini Pro 2—approach human performance in specific contexts, notable gaps remain, especially in recognizing negative affect conveyed through bodily gestures. These findings underscore the promise of GAI in socially embedded applications such as clinical support, while highlighting the need for continued development using ecologically valid, diverse, and multimodal datasets to enhance the emotional competence of artificial agents.

**Disclosure of Interest:**

None Declared

## Long descriptions

### Image 1: Long description

A bar chart titled Mean Proportion of Correct Emotion Recognition (%) - Body Gesture and Voice. The y-axis represents the percentage from 0 to 100. The x-axis lists three models: Pro 1.5, Pro 2.0, and Flash 2.0. Two horizontal dashed lines represent human benchmarks: a blue line for Human Body Gesture at approximately 76% and a red line for Human Voice at approximately 45%.

For each model, two bars are shown: blue for Body Gesture (Model) and orange for Voice (Model).

* Pro 1.5: Body Gesture is 60.14% and Voice is 35.71%.

* Pro 2.0: Body Gesture is 71.39% and Voice is 47.14%.

* Flash 2.0: Body Gesture is 64.72% and Voice is 45.36%.

Pro 2.0 and Flash 2.0 exceed the human benchmark for voice, while all models remain below the human benchmark for body gesture recognition.

Navigate back to Image 1:.

### Image 2: Long description

The y-axis represents the Mean Proportion of Correct Recognition percentage from 0 to 100. The x-axis lists three models: Pro 1.5, Pro 2.0, and Flash 2.0. A legend on the right identifies blue bars as Positive Model, orange bars as Negative Model, a blue dashed line as Positive Human, and a red dashed line as Negative Human. Both human baseline lines are positioned horizontally at approximately 76 percent.

Data points from left to right:

* For Pro 1.5, the blue bar is at 64.29 percent and the orange bar is at 53.25 percent.

* For Pro 2.0, the blue bar is at 85.36 percent and the orange bar is at 58.75 percent.

* For Flash 2.0, the blue bar is at 79.64 percent and the orange bar is at 50.75 percent.

Across all models, recognition of positive emotions is significantly higher than negative emotions. Only Pro 2.0 and Flash 2.0 exceed the human baseline for positive emotion recognition, while all models perform below the human baseline for negative emotion recognition.

Navigate back to Image 2:.

### Image 3: Long description

A bar chart titled Mean Proportion of Correct Positive/Negative Emotion Recognition (%) - Voice.

Axes and Legend:

* Y-axis: Mean Proportion of Correct Recognition (%) ranging from 0 to 100.

* X-axis: Three model categories: Pro 1.5, Pro 2.0, and Flash 2.0.

* Legend: Blue dashed line for Positive (Human), red dashed line for Negative (Human), blue solid bar for Positive (Model), and orange solid bar for Negative (Model).

Reference Lines:

Two horizontal dashed lines represent human performance benchmarks across the entire chart. The red dashed line (Negative Human) is slightly higher than the blue dashed line (Positive Human), both positioned between the 40 and 50 percent marks.

Data Groups (Left to Right):

* Pro 1.5: Positive (Model) is 35.71% and Negative (Model) is 38.46%. Both are below the human benchmarks.

* Pro 2.0: Positive (Model) is 52.85% and Negative (Model) is 43.85%. Positive recognition exceeds human benchmarks, while negative recognition is slightly below.

* Flash 2.0: Positive (Model) is 56.78% and Negative (Model) is 42.69%. Positive recognition is the highest in the chart, while negative recognition remains below human benchmarks.

Navigate back to Image 3:.

